# Nursing under the Poor Law

**Published:** 1913-04-05

**Authors:** 


### NURSING UNDER THE POOR LAW.

Retrospection is seldom encouraging or cheerful, but our points will be more clear in this introductory article if we note some facts in the past history of Poor-Law nursing; and as a key-note we should bear in mind that the one drastic change in the administration of Poor-Law institutions was the separation in the Metropolis of the infirmaries from the workhouses, and that this was effected by the Act of Parliament passed March 29, 1867.

The "patching-up" method which has been adopted by the Local Government Board almost entirely of late years will never give any satisfactory or complete system; it is mere waste of time, and after each effort on the part of the authorities matters remain still unsettled and open to criticism. We want another sweeping reform to deal with the question of the sick inmates and the nursing of the smaller workhouse infirmaries. No wholly effectual reforms have followed the taking of evidence, the issuing of reports, the framing of "recommendations," during the past fifteen or sixteen years, although certain improvements have been effected. These, however, are not universal, because they are not compulsory. It would be a matter for supreme dissatisfaction if again the deliberations of a Departmental Committee should end in a fiasco similar to that of the Report of the Departmental Committee issued in 1902. It will be remembered that important and expert evidence was taken on that occasion, and yet amongst the recommendations made was that of the establishment of minor training schools and the creation of the "qualified nurse," who was intended to be a special asset of Poor-Law administration to meet the difficulty of securing an adequate supply of nurses for service in rural workhouse infirmaries. This suggestion was fortunately quashed by the activity of the Workhouse Nursing Association, which was instrumental in procuring wide support to a strong memorial addressed to the President of the Local Government Board; and little more was heard of the Departmental Committee's recommendations, which were the result of months of work.

### Pioneers of Reform.

In the past pioneers have broken up the ground; many individuals holding official positions have done excellent work in their own special institutions; and voluntary effort has stimulated public opinion. The results have been certain improvements, notably the appointment of women Guardians, which has had far-reaching effect for good; the appointment of women inspectors by the Local Government Board after many years of weary waiting; and the issue of the Nursing Order of 1897, which insisted upon the appointment of a fully-trained superintendent nurse in those workhouse infirmaries where three or more nurses are employed. All these are important steps in advance; but no standard of training for other nurses was demanded by this Order, and therefore it has proved ineffectual in ensuring any proper system of trained nursing in all institutions. These latter-day improvements make us almost forget the bygone years when the late Miss Louisa Twining was first inspired to undertake what might be truly called her life work for the good of humanity, when in 1853 she first visited a poor woman in the Strand Union Workhouse, and her sympathies were aroused by the lamentable condition of the sick inmates. She was the very first visitor from the outside of the well-to-do class, and she only gained admission as a friend of the old woman in whom she was interested. We know how cautiously the doors of these places have been opened to visitors, and Miss Twining fought for five years before she obtained official sanction to any organised system of visiting by others besides the relatives and friends of the inmates. For years the Local Government Board declined to give its sanction without the approval of Boards of Guardians, who in their turn objected on the plea of "interference" or "divided management." Happily those days have passed, and we do not hear of the "deeds of despotism and cruelty" allowed by a system managed, without criticism and the light of public opinion, by Guardians with no special knowledge and officers equally unskilled, and "sanctioned or perhaps ignored by those who appointed or upheld them."

However, we still hear of gross neglect of patients by ignorant and incompetent untrained officials, who, though appointed as nurses, are in no way trained or qualified to nurse, of sad accidents as the result of an insufficient staff, and of an absence in many places of any system of night nursing. So recently as the summer of 1912 in a workhouse nursery the children were tied up in chairs for three or four hours at a time when they could not go out; as many as nine or ten were bathed in the same water; part of the food apportioned to them was confiscated after it reached the nursery, thus rendering them ill-fed, and when fretful they were given bromide. We could quote many instances to show how unequal are the methods of treating our sick and helpless. This must be obviously the case when no uniform system is demanded by headquarters, and while so many details are left to the conflicting opinions and various standards of Boards of Guardians composed of all sorts and classes of men and women, to whom sickness and the nursing of the sick still remain only a part of poor relief, and not a special department requiring skilled and expert care.

The forcible and courageous pioneers, who have passed away, and do not, so far, seem to be replaced by others of equal vigour, finished a great work, though to themselves they seemed to have made sadly little way. The age of any pioneer movement does not come twice. The natural sequence of ardent enthusiasm and personal influence should be a practical handling of the question, with full knowledge of its needs. The wise suggestions of these earlier workers have been preserved, and it is noteworthy that they practically agree with the views of reformers of the present time. The late Dr. Sheen, of Cardiff, the late Dr. Dolan, of Halifax, besides Miss Twining and Sir William Bousfield, to name only a few, have left a record of their views which we venture to think are founded on the same facts and contain the same suggestions for reform as those which prominent workers of to-day long to enforce.

We have, too, the valuable information gained by evidence taken by the Royal Commission on the Poor Laws, whose Reports have not yet been acted upon; in fact, we do not lack the basis of facts and expert opinion, extending over more than fifty years. There are persons, doubtless, who see by knowledge and experience what the present system lacks. We certainly know to-day that it is in the best interests of all concerned that the sick should be made well if possible, and as quickly as possible, whether they be rich or poor. It is economy; it is hygienic as regards the community; and, above and beyond all, it is humanity. In these days of enlightenment, besides the obvious advantage and comfort to the patients themselves, and the avoidance of needless suffering in chronic and helpless cases, we recognise the practical economy which follows skilled medical care and nursing of certain cases, both as regards duration of residence in the sick wards and the restoration to health and activity of the breadwinner or of the mother of a young family. We see even beyond this, in the exercise of preventive measures unknown to untrained nurses, the arrest, maybe, of incipient disease, which is of the highest importance, and the consequent building up of a healthier and more physically fit nation.

We are not dealing in this article with suggestions of methods to be adopted to enforce a uniform and workable, as well as competent, system; but we would give a hint to the Local Government Board that it is only just to the public that the authorities should seek expert consultation and discussion on the many questions of detail in settling their new Order, a draft of which is now under consideration. It is a crisis in Poor-Law history. The Nursing Order of 1897 was the first issued for fifty years, and is now nearly sixteen years old; it is hardly likely that another official pronouncement of the kind will be made for many years. It appears that, contrary to usual custom, no evidence has been taken by the Departmental Committee before drafting the Order, that it is held as confidential, and that copies have been distributed for criticism only in a very limited sphere. We have, however, in the extracts published by the press, sufficient information to show some glaring imperfections, and we again urge caution before official sanction is given to the Order.

For instance, it would be a disaster if the recommendation were adopted in reference to a continuance of the attendance of paupers in the sick wards, which the draft Order marks a precedent by suggesting. Hitherto this retrograde custom has been denounced, even though circumstances seemed to excuse its adoption in a modified way, and it is astounding that such a clause as the following should emanate from the central authority. It rightly has raised a storm of indignation amongst those who have studied and understand those matters. The recommendation is that the master may direct that an inmate be employed "in the sick wards, lunatic wards, or nurseries . . . if approved for the purpose by the medical officer, and acting under the immediate supervision of a paid officer." There is no condition that the "paid officer" should be a trained nurse even; and the suggestion is entirely retrograde, and would be a loophole for grave abuses.

It must be remembered in this connection that in a small workhouse the medical officer is not resident, and is frequently, if not usually, a hard-worked country doctor, to whom even a daily visit is difficult and often impossible.

It is significant that the only reform that we can point to as perfectly carried out was the outcome of the Bill brought in by Mr. Gathorne-Hardy (Lord Cranbrook), referred to in our first paragraph, for the separation of the Metropolitan infirmaries from the workhouses. This, because it was an Act of Parliament, had to be accepted. It was the result of much serious and able effort, which brought the *Lancet* Commission with its incontrovertible facts and details, and year by year we have seen these infirmaries improve, having the stability of the unassailable action of the authorities. Many other efforts have certainly been for good, and have advanced the question, but they have given no perfect system.

In the summer of 1908 Mr. Asquith and Lord Haldane both spoke on the question. Mr. Asquith prophesied that "in twelve months, or at most two years, the existing distinctions and classification of our thoroughly unscientific, and in some respects inhumane, Poor-Law system would be completely remodelled." Lord Haldane considered that "the helpless, the sick, the insane, the children who were not looked after by their parents, were the subjects for a curative policy, and ought to be treated in institutions wholly different from the class who were condemned to the workhouse." It is nearly five years since these two statesmen spoke thus; and at the time it was hoped that their utterances referred to what may have been expected from the Reports of the Royal Commission.

Therefore, we recur to our first two paragraphs, and would reiterate: Nothing short of drastic reforms reached after consultation with men (especially medical men) and women who know the conditions, and whose care is for the nation's health, and the reduction of unnecessary suffering to the helpless poor, will meet the case. The Local Government Board is delaying a question which requires serious, thorough, and expert consideration, and for which there is a remedy which can and must be found. It is unthinkable that the labour and the cost of a Royal Commission can, even after a lapse of years, be allowed to become a dead letter.

